# Seryl phosphorylation modulates chaperonin function of Mycobacterial chaperonin, Cpn60.1

**DOI:** 10.1016/j.cstres.2026.100220

**Published:** 2026-09-25

**Authors:** C. M. Santosh Kumar, Monika Singh, Shekhar C. Mande, Peter A. Lund

**Affiliations:** 1 School of Biosciences & Institute of Microbiology and Infection, University of Birmingham, Birmingham B15 2TT, UK; 2 Laboratory of Structural Biology, Centre for DNA Fingerprinting and Diagnostics, Hyderabad 500001, India; 3 Laboratory of Structural Biology, National Centre for Cell Science, Pune 411007, India

**Keywords:** Chaperonins, GroEL, Mycobacteria, Phosphorylation-switch, Complementation, Normal mode analysis

## Abstract

The GroEL chaperonin forms a 14‑subunit double ring that encapsulates client proteins for folding with the aid of the cochaperonin GroES. Mycobacteria encode two GroEL homologs: Cpn60.2 (which is essential) and Cpn60.1 (which is non-essential but which has been proposed to have roles in pathogenesis). Cpn60.1 homologues exhibited impaired oligomerization and thereby failed to exhibit chaperonin activity. We previously showed that phosphorylation of MtCpn60.1 S393 correlates with oligomeric assembly. Here, we show that canonical oligomerization residues are replaced by serines in Cpn60.1 homologues, enabling a phosphorylation‑switch. We generated phospho-mimetic variants at these positions and assessed their ability to functionally replace *Escherichia coli* GroEL. Using functional complementation and serial-depletion assays, we demonstrate that although the wild-type MtCpn60.1 failed, a double phospho-mimetic variant (S393D + S75D) exhibited chaperonin activity in *groEL* conditional mutant strains but did not functionally replace GroEL in a *groEL* knock out strain. Further, native PAGE analysis of the S75D/S393D double phospho-mimetic variant was consistent with higher-order oligomerization, while mass spectrometry of MtCpn60.1 confirmed S75 phosphorylation and identified additional phosphoserine-containing peptides. Homology modeling suggests that phosphorylation may alter a residue-pair interaction involved in oligomerization. Together, these findings support a model in which phosphorylation contributes to higher-order oligomerization and partial chaperonin activity of Cpn60.1.

## Background

Prokaryotic chaperonin genes are stress inducible,^1^, ^2^ often essential^3^, ^4^, ^5^, ^6^ and highly conserved.^7^, ^8^, ^9^, ^10^ Current understanding of the functional and structural aspects of chaperonins is largely based on studies on *Escherichia coli* GroEL and GroES.^1^, ^11^, ^12^, ^13^, ^14^ The larger component of this system, GroEL, assembles into a tetradecameric double toroid,^15^, ^16^, ^17^ enclosing two isologous cavities that encapsulate client proteins, while the smaller component, GroES, forms a seven-membered lid for one of these cavities.^18^, ^19^, ^20^, ^21^ The double toroid architecture is essential for productive folding of stressed or naive client proteins with exposed hydrophobic patches that are otherwise buried in their native conformation.^1^, ^12^, ^22^, ^23^ The client proteins are bound by and encapsulated into one of the chaperonin cavities, classically known as Anfinsen cages.^24^, ^25^, ^26^ These client proteins can then fold in the sequestered environment of the cavity, capped by the GroES. Binding of ATP to the other ring in the complex triggers a conformational change in the client-bound ring, thereby releasing the client proteins in their folded form.^8^, ^27^, ^28^ This ATP-mediated binding and hydrolysis cycle thus enables client protein folding.

Although most bacteria have one copy each, the chaperonin and co-chaperonin encoding genes, usually expressed as single operon, many bacterial species encode multiple chaperonin homologues.^3^, ^8^, ^29^, ^30^ The multiple chaperonin genes were first identified in *Mycobacteria*.^31^ *Mycobacterium tuberculosis* encodes two stress-inducible chaperonin homologues, Cpn60.1 and Cpn60.2.^32^, ^33^, ^34^ These proteins exhibit significant deviations from canonical chaperonin characteristics. For example, although Cpn60.2 is essential^6^ and can functionally replace *E. coli* GroEL,^35^, ^36^ it assembles as a lower oligomer when produced in *E. coli*.^37^ Further, Cpn60.2 assembles as a tetramer in *M. tuberculosis* but gets destabilized and dissembled into a monomer upon N-terminal proteolytic cleavage by an envelope-associated serine protease, Hip1.^38^ While the resulting cleaved Cpn60.2 monomer stimulates macrophage cytokine secretion,^38^ the full-length tetramer elicits dendritic cell proinflammatory responses.^39^ However, Cpn60.1 is dispensable in *M. tuberculosis* and other Mycobacterial species,^6^, ^40^, ^41^ fails to assemble into the characteristic tetradecamers under standard conditions^3^, ^8^, ^37^, ^42^ and consequently fails to exhibit chaperonin activity in vitro^37^ or in vivo.^36^, ^43^ Cpn60.1 has been shown to be secreted,^44^ involved in maintaining bacterial membrane integrity against redox stress^41^ and has a role in the Crabtree effect and assembly of the FASII complex, leading to biofilm formation.^45^, ^46^ It can also stimulate monocyte proinflammatory cytokine synthesis,^47^, ^48^ and inhibit the activity of osteoclasts and hence the breakdown of bone matrix.^49^ It is required for granuloma formation in mouse and guinea pig models^6^ of tuberculosis.

Structural comparison of Cpn60.1’s apical domain suggested that Cpn60.1 is likely to exhibit GroEL-like client protein interactions.^50^ Further, Cpn60.1 prevented aggregation and exhibited limited ability to assist the folding of the obligate chaperonin clients, DapA, FtsE, and MetK.^51^ These studies suggested that Cpn60.1 can bind its client proteins effectively and assist folding partially. As Cpn60.1 exhibits impaired oligomerization, we investigated if oligomerization resulted in partial function. Sequence and structural analysis of mycobacterial chaperonins show that several residues that form salt bridges in GroEL are mutated either to serine or threonine in Cpn60.1, but not in Cpn60.2.^37^, ^52^ We examined this by aligning Cpn60.1 and Cpn60.2 sequences and observed that several salt-bridge-forming residues at the inter-subunit interfaces of GroEL and Cpn60.2 homologues are replaced by Serine or Threonine residues in Cpn60.1 (Table S3 and Supplementary data file 1). Further, *M. tuberculosis* encodes 11 eukaryotic-like serine threonine protein kinases (STPKs)^53^ and is known to use phosphorylation to regulate the function of multiple proteins important in pathogenesis.^54^, ^55^, ^56^, ^57^, ^58^ These observations suggest a possible role for phosphorylation in Cpn60.1 oligomerization and hence chaperonin function. Surface phosphorylation-modulation of chaperonin activity has been observed in *Bacillus anthracis* and *Staphylococcus aureus*.^59^ *M. tuberculosis* Cpn60.1 was shown to be phosphorylated *in vitro* on two threonine residues by the Mycobacterial STPK, PknF.^60^ Serine phosphorylation was also detected in vivo in the tetradecameric form of Cpn60.1 isolated from *M. tuberculosis*,^43^ leading to the hypothesis that serine phosphorylation may regulate the oligomerization and possibly the chaperonin activity of Cpn60.1.^42^

Tetradecameric Cpn60.1 was demonstrated to be phosphorylated on S393.^43^ As this site is in the putative intermediate domain, while the inter-subunit interface responsible for tetradecameric assembly is in the equatorial domain,^14^, ^16^, ^61^ this raised the possibility that other residues, potentially in the equatorial domain, might also be phosphorylated and hence affect oligomerization and chaperonin function. We have therefore investigated the potential effect of phosphorylation on the function of Cpn60.1 by using phospho-mimetic Cpn60.1 variants of other serine residues located in its putative equatorial domain, using a complementation assay to assess functionality. Phosphorylation on these residues in the native protein was studied using mass spectrometry, and the oligomeric nature of the phospho-mimetic variants was assessed using native polyacrylamide gels. Homology models of Cpn60.1 were built to corroborate the potential effect of phosphorylation on Cpn60.1’s oligomerization and activity.

## Results

### Cpn60.1 homologues exhibit distinct conservation profiles at the oligomerization interface

To understand the differences in oligomerization properties of MtCpn60.1 and MtCpn60.2, we examined 58 Mycobacterial Cpn60.1 and Cpn60.2 homologue polypeptide sequences (retrieved from UniProt) and their phylogenetic relationships inferred using a neighbor-joining algorithm. Cpn60.1 and Cpn60.2 homologues clustered into two different clades of the polynomial-time phylogenetic tree; Cpn60.1 homologues exhibited higher divergence (61% branches with ∼ 0.9 phylogenetic distance) than Cpn60.2 homologues (29% branches with ∼ 0.9 phylogenetic distance) (Figure 1a) and showed distinct conservation at various key residues; the residues in ATP-biding pseudo-Walker motif residues were conserved in both Cpn60.1 and Cpn60.2 homologues, and the residues involved in inter-subunit interactions showed lower conservation (Table 1 and Supplementary data file 1), suggesting that the Cpn60.2 homologues, largely being the essential housekeeping chaperonins, encounter higher selection pressure and hence exhibit lower divergence.^62^, ^63^

**Fig. 1:**
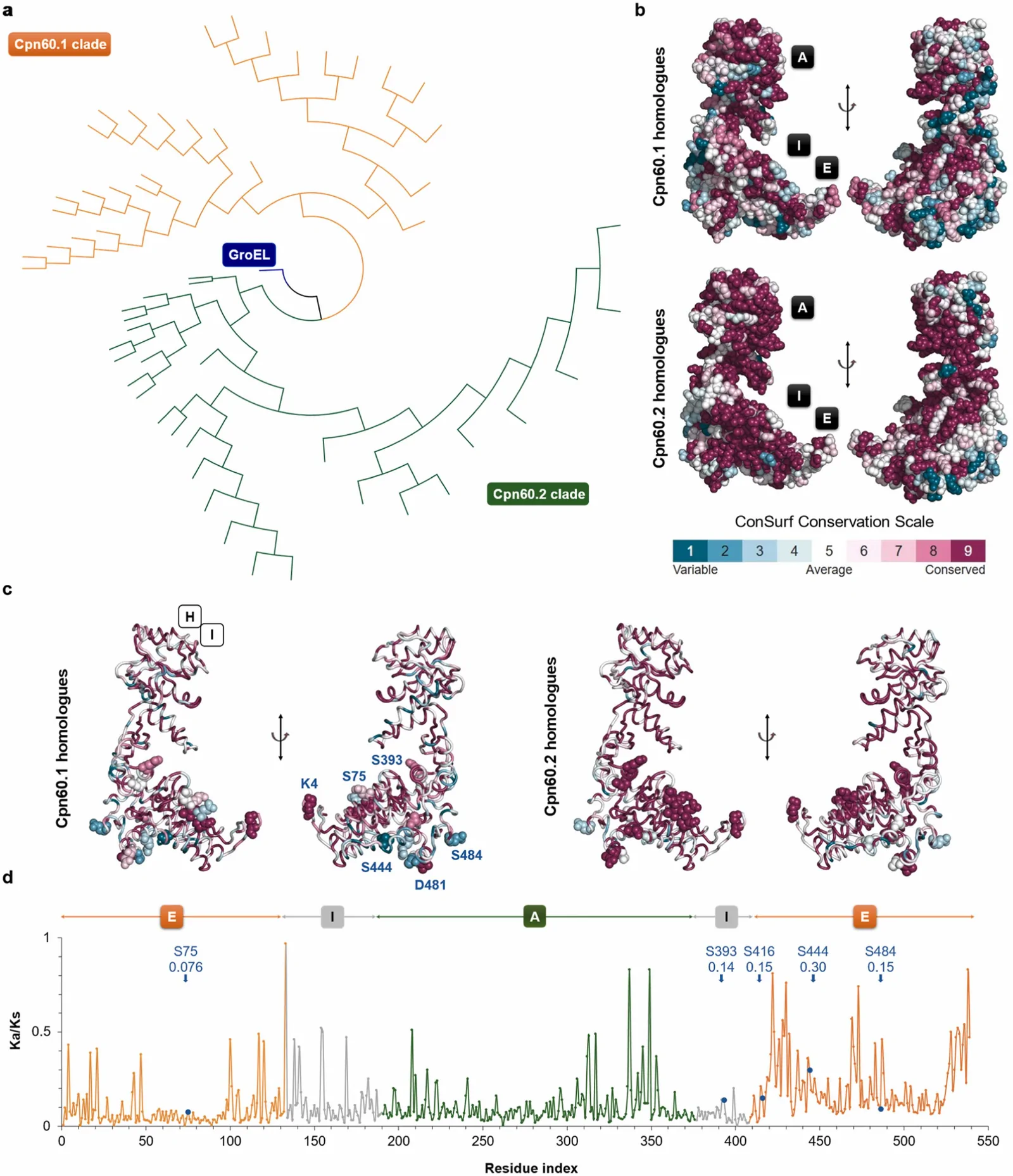
*Cpn60.1 homologues exhibit distinct conservation profiles.***(a)***Mycobacterial* Cpn60.1 and Cpn60.2 homologue sequences (58) were retrieved from UniProt and were aligned to *E. coli* GroEL (please see Supplementary data file 1 for the alignment). The phylogenetic relations were assessed using a neighborhood-joining algorithm in MEGA 11 and presented as a phylogenetic tree. The multiple sequence alignment of the chaperonin genes used in this analysis and a detailed phylogenetic tree showing phylogenetic distances is provided in Supplementary data file 1. **(b)** The Cpn60.1 and Cpn60.2 homologues were aligned separately, and the resulting MSAs were used to calculate the site-specific conservation rates using ConSurf’s Rate4Site algorithm^64^ and mapped onto *E. coli* GroEL sequence structure (1AON – Chain A^20^). The letters A, I and E indicate the putative apical, intermediate, and equatorial domains, respectively. **(c)** Conservation of inter-subunit interactions in Cpn60.1 and Cpn60.2 interactions. The putative interacting residues are shown as spheres and listed in Table 1. Key residues are labeled. Letters H and I in open boxes indicate the helices H and I, respectively. **(d)** Codon-level conservation of MtCpn60.1 was inferred using a ratio of synonymous (*K*_s_) and nonsynonymous (*K*_a_) substitutions, and the resultant ratios are plotted as a function of MtCpn60.1 residue index. Blue-colored arrows indicate the potential phosphorylating serine residues and their *K*_a_/*K*_s_ ratios.

**Table 1:** Amino acids that are potentially involved in inter-subunit interactions of Cpn60 homologues.

Residues in GroEL	Corresponding residues in Cpn60.1 homologues	Corresponding residues in Cpn60.2 homologues
K4	K (100%)	K (100%)
E59	E (80%), D (20%)	E (100%)
I60	I (100%)	I (100%)
E61	D (63%), E (36%)	E (100%)
L62	L (100%)	L (100%)
E76	S (96%), E (3%)	E (100%)
R197	K (96%), R (3%)	K (96%), R (3%)
R284	R (100%)	R (100%)
R285	R (100%)	R (100%)
E386	E (96%), D (3%)	E (100%)
R395	S (63%), A (30%), R (6%)	R (100%)
V417	S (50%), A (43%), V (6%)	V (100%)
L444	S (60%), A (26%), F (3%), T (3%), E (3%), L (3%)	R (66%), K (30%), L (3%)
R452	Y (73%), F (23%), R (3%)	K (96%), R (3%)
E461	D (96%), E (3%)	E (100%)
S463	S (50%), A (46%), H (3%)	G (96%), S (3%)
V464	V (100%)	V (100%)
E484	S (33%), E (33%), T (26%), G (3%), D (3%)	E (63%), V (33%), D (3%)

Numbers in the parentheses indicate the variability frequency at the amino acid positions.

To test if the principal variation was in the inter-domain regions, we aligned the full-length sequences of the Cpn60.1 and Cpn60.2 sequences separately with *E. coli* GroEL and calculated the residue-specific divergence rates of Cpn60.1 and Cpn60.2 homologues, using the rate4site algorithm^64^ and EMBOSS Cons. We mapped the conservation color scales (1−9) onto *E. coli* GroEL structure (Figure 1b). The Cpn60.1 homologues showed overall higher divergence than the Cpn60.2 homologues (Figure 1b), with 18.25% and 10.04% of the sites in Cpn60.1 and Cpn60.2 homologues showing conservation scores above 0.9 (Figure S1 and Supplementary data file 2), suggesting higher divergence in Cpn60.1 homologues. Further, Cpn60.1 and Cpn60.2 homologues showed mean diversity of 0.29 +/− 0.18 and 0.15 +/− 0.03, in agreement with the higher diversity among Cpn60.1 homologues. Regions of the putative substrate binding helices H and I, intermediate domains, pseudo-walker motif (ATP-binding residues), and inter-domain boundaries were conserved in both Cpn60.1 and Cpn60.2 homologues (conservation scales close to 9) (Figure S1 and Supplementary data file 2). As the majority of the regions involved in inter-subunit interactions and oligomerization exhibited larger divergence among Cpn60.1 homologues (conservation scales between 1 and 5) than in Cpn60.2 homologues (conservation scales between 8 and 9) (Figure S1c and Supplementary data file 2), we mapped the residues involved in the putative inter-subunit interactions (Table 1) onto the *E. coli* GroEL structure (Figure 1c). These residues exhibited greater conservation in Cpn60.2 homologues than in Cpn60.1 homologues (Figure 1c and Supplementary data file 2), suggesting a potential link between this divergence and impaired oligomerization. The EMBOSS Cons, which derives consensus sequence from an MSA, also showed lower conservations of the interface residues (Figure S2), in agreement with the Rate4Site analysis.

Therefore, we investigated if any of these sites are potentially phosphorylated using the standard phospho-prediction servers, NetPhos 3.1 and KinasePhos 2.0 (Figure S3). NetPhos algorithm predicted phosphorylation on S75, S393, S444, and S484 (Figure S3a), while KinasePhos predicted phosphorylation on S75, S393, and S416 (Figure S3b). Interestingly, these serines lie at the putative inter-subunit interface within the heptameric ring (S75, S416, and S484) and between the two rings (S444).^12^, ^16^, ^61^ Further, these sites are modified from different residues in *E. coli* GroEL and Cpn60.2 into serines in Cpn60.1 paralogues (Supplementary data file 1), other predicted serine sites were synonymously substituted in GroEL and Cpn60.1 homologues. Therefore, we investigated the serine residues (S75, S393, S416, S444, and S484) further.

As Cpn60.1 has been shown to exhibit chaperonin activity in folding specific client^45^ or model proteins,^51^ we wished to understand the unusual evolution of these residues. We estimated the rate of evolutionary change in MtCpn60.1, using the corresponding coding nucleotide sequences of cpn60.1 homologues, and calculated the ratio of nonsynonymous substitutions per nonsynonymous site (Ka/Ks) for each codon to estimate the purifying selection, using the MEGA Phylogenetic analysis application. The resulting ratios were mapped on to the sequence index (Figure 1d). The probable phosphoserine residues (S75, S393, S416, S444, and S484) showed lower ratios (0.076–0.3) suggesting a strong purifying (negative) selection on these residues (Figure 1d) which implies some functional significance. As these residues are changed to serines in Cpn60.1 paralogues (Supplementary data file 1) and their phosphorylation has been predicted (Figure S3), we explored whether phosphorylation of these serines was functionally significant, for example, by facilitating oligomeric assembly.

### The S75D/S393D double phospho-mimetic variant exhibits partial chaperonin activity

To study the effect of serine phosphorylation on chaperonin activity, we generated site-specific phospho-mimetic (serine to aspartic acid) mutations at different serine residues in MtCpn60.1 and used complementation to assess the ability of the resulting variants to replace *E. coli* GroEL function in vivo. Candidate residues were identified using a multiple sequence alignment of mycobacterial Cpn60.1 and Cpn60.2 chaperonin paralogues (Figure 1 and Supplementary data file 1). In addition to S393, which we had previously identified as being important for oligomerization of MtCpn60.1^43^ (Figure S4), we chose four additional serines, S75, S416, S444, and S484 (Figure 2a). We mutated these serines in MtCpn60.1 to aspartic acid, which mimics a phosphorylated serine, both structurally and chemically.^65^ The ability of these phospho-mimetic MtCpn60.1 variants to functionally replace *E. coli* GroEL was assessed using *E. coli* LG6, a *groE* conditional mutant strain, in which the chromosomal copy of the *groE* operon is under the control of D-lactose/IPTG-inducible *P*_*lac*_ promoter.^66^ Since *groEL* and *groES* are essential, if the *P*_*lac*_ promoter is repressed, this strain grows only when a functional chaperonin and co-chaperonin are provided in *trans*. Therefore, the genes expressing chaperonin variants (GroEL or MtCpn60.1 and the phospho-mimetic variants) were cloned along with their cognate co-chaperonin genes (either GroES or Cpn10, respectively) in operonic arrangement (Table S1) for co-expressing cognate chaperonin and co-chaperonin pairs. In these complementation experiments, the MtCpn60.1 S393D variant showed a low level of chaperonin activity, consistent with our previous results, while the other four variants showed no activity (Figure 2b). To test whether combining one or more of the other four phospho-mimetic mutations with the MtCpn60.1 S393D variant might improve its chaperonin activity, we generated four double phospho-mimetic mutations in the S393D variant (MtCpn60.1 S393D/S75D, S393D/SS416D, S393D/SS444D, and S484D/S393D), and the ability of these double mutants to function was again assessed in LG6. One of the double mutants, MtCpn60.1 S75D/S393D (here after known as DM), complemented in LG6 at 30 °C and 42 °C (Figure 2c), while the wildtype and the other double mutants (MtCpn60.1 S393D/SS416D, S393D/SS444D, and S484D/S393D) were non-functional. As the S75D single mutant failed to complement, whereas the S75D/S393D double mutant complemented LG6, these data indicate that the two substitutions together increase chaperonin activity. However, the double mutant remains only partially functional and does not fully replace GroEL. We also investigated the ability of the DM variant to complement in *E. coli* MGM100, a strain where the chromosomal *groE* operon is under the control of the L-arabinose inducible *P*_*BAD*_ promoter.^67^ The DM variant complemented this strain at 30 °C but not at 42 °C (Figure 2c).

**Fig. 2:**
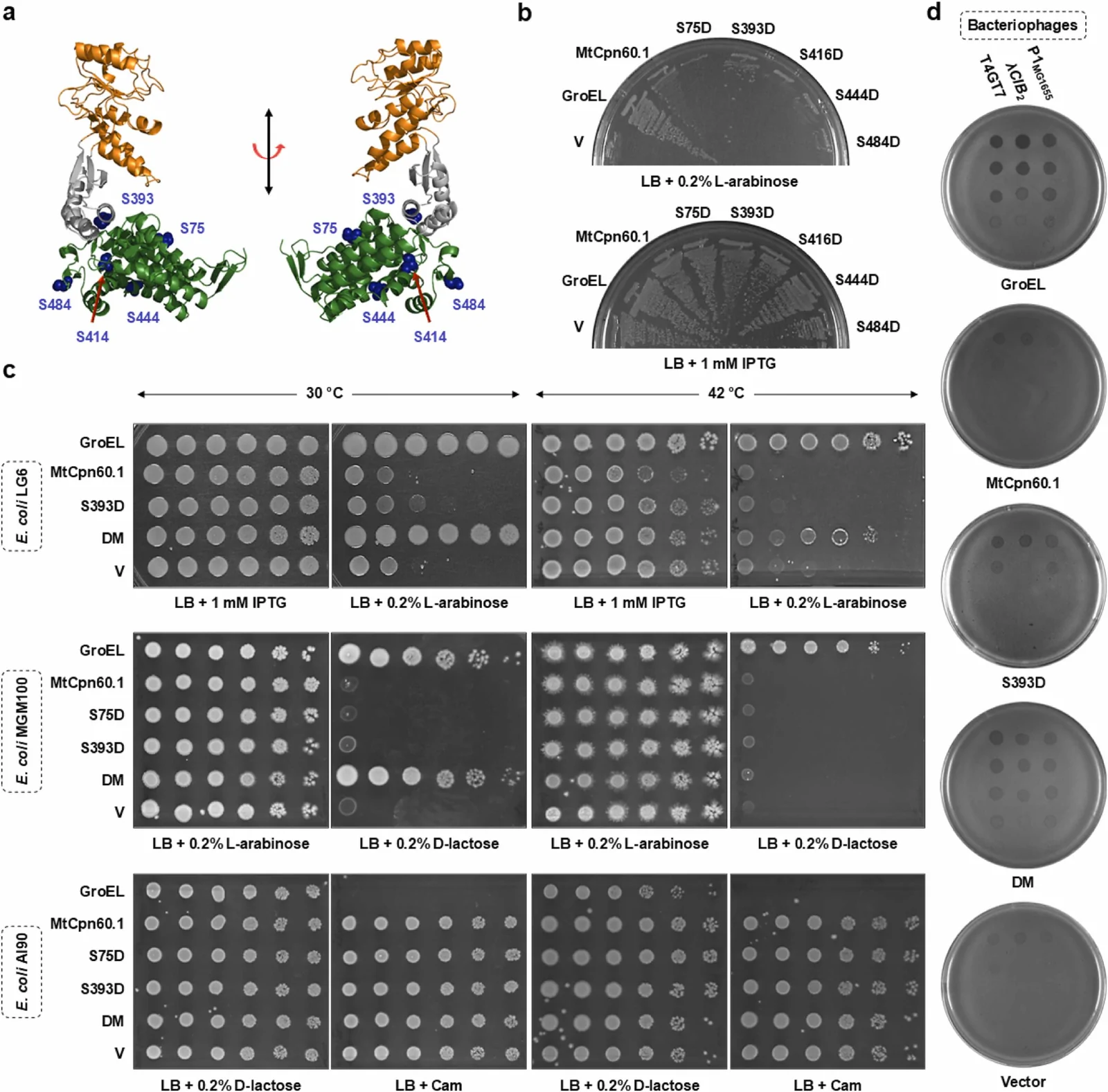
*Serine phospho-mimetic mutations in MtCpn60.1 enable partial chaperonin activity.***(a)** Homology model of MtCpn60.1 indicating the serine residues that were mutated to aspartic acid to mimic phosphorylated serines (blue spheres). The predicted apical, intermediate, and equatorial domains of MtCpn60.1 are colored in gold, silver, and green, respectively. The molecular figures were generated using Pymol 2.0. **(b)***E. coli* LG6 cultures expressing the chaperonin variants were streaked on LB agar plates supplemented as indicated, and the plates were incubated at 30 °C. S75D, S393D, S416D, S444D, and S484D indicate the respective MtCpn60.1 phospho-mimetic mutants. V indicates the vector-only control. **(c)***E. coli groEL* mutant strains LG6 and AI90 were transformed with pBAD24-derived plasmids expressing the indicated chaperonin genes, while MGM100 was transformed with pTrc99A-derived plasmids expressing the same genes. Serially diluted cultures of the different *E. coli groEL* mutant strains expressing the indicated chaperonin genes were spotted onto LB agar plates, supplemented as shown. The plates were incubated at the indicated temperatures. S75D and S393D refer to the respective *M. tuberculosis* Cpn60.1 phospho-mimetic mutants, while DM refers to the S75D and S393D double mutant. Cam: 25 µg/mL Chloramphenicol. **(d)** Lawns of *E. coli* LG6 expressing the different chaperonin genes shown were prepared on LB agar plates supplemented with 0.2% L-arabinose. Serial dilutions of the different bacteriophages were spotted onto the lawns, followed by incubation at 30 °C.

This difference in the behavior of the DM variant in LG6 and MGM100 suggests that the DM variant exhibited only partial chaperonin function, with the different levels of residual GroEL in these strains under non-induced conditions leading to different levels of overall chaperonin function in the *E. coli* cells. The *P*_*lac*_ promoter, which regulates the *groE* operon in *E. coli* LG6 is more leaky than the *P*_*BAD*_ promoter in *E. coli* MGM100.^67^ Consequently, the amount of residual GroEL in *E. coli* LG6 under non-inducing conditions will be higher than in MGM100. This higher level might enable GroEL in *E. coli* LG6 to assist either folding or assembly of MtCpn60.1 at 42 °C or might assist in folding of essential clients that cannot be folded by the DM variant at this temperature. To study the potential function of MtCpn60.1 S75D/S393D, we used two strains: the *E. coli groEL* deletion strain AI90, in which the chromosomal copy of *groEL* is insertionally inactivated using a Kan^R^ cassette and *groEL* is provided from a Cam^R^ plasmid,^68^ and *E. coli groEL*^*ts*^ strain SV2, which carries the *groEL44* allele.^2^ The Cam^R^ plasmid in AI90 carrying *groEL* can only be lost if a functional chaperonin is provided from a different plasmid; in this event, the strain will become Cam^s^. In *E. coli* SV2, *groES* and *groEL* are expressed from the natural promoter. Therefore, this strain produces the natural levels of the GroEL variant, which due to the *Ts* mutation, fails to support growth above 42 °C, unless a functional chaperonin is provided.^2^ We observed that the DM variant did not support growth of AI90 in the absence of the shelter plasmid-borne GroEL (Figure 2c) but could rescue the temperature-sensitive phenotype of SV2 (Figure S5). This observation is consistent with the model that the DM variant exhibits partial chaperonin function, not as complete as that of GroEL but greater than that exhibited by the wild type MtCpn60.1 or its S393D variant. The interpretation of these results is considered further in the Discussion.

As the development of bacteriophages lambda, P1 and T4 requires a functional GroEL/ES system,^69^ we investigated if the DM variant could support phage morphogenesis. While strains expressing either the wild-type MtCpn60.1 or the single mutants S393D could not support bacteriophage development in LG6, that expressing the DM variant could support the bacteriophage development (Figure 2d).

### The DM variant exhibits partial chaperonin activity *in vivo*

To further characterize the partial activity of the Cpn60.1 variants, we monitored the growth in liquid culture of *E. coli* MGM100 strains producing either GroEL, MtCpn60.1, MtCpn60.1 S75D, MtCpn60.1 S393D, or DM along with their cognate co-chaperonins under restrictive conditions (in the presence of 0.2% D-glucose and absence of lactose and arabinose). In the absence of the inducer L-arabinose, the levels of GroEL and GroES expressed from the chromosome will become depleted by growth and dilution, eventually becoming limiting for growth unless complementing activity is supplied in trans.^68^ The extent to which strains can continue growing following cycles of growth and dilution can thus be taken as a measure of in vivo chaperonin activity. Cultures without any chaperonin provided in trans (the vector-only cultures) did not show significant growth in the restrictive conditions, whereas cultures with GroES and GroEL supplied in trans were able to continue growing at normal rates after two cycles of growth and dilution (Figure 3). Strains expressing wildtype MtCpn60.1 + MtCpn10 supported growth partially only in the first round of culturing, and those expressing MtCpn60.1 with either the S75D or S393D single mutations showed better growth (reaching a higher OD_600_) in the first round. Strains expressing the DM variant supported growth for the first two rounds of culturing, but no further growth was seen following dilution a third time. These results show that the DM variant exhibited greater chaperonin activity in vivo than either of the single mutants, which in turn are more active than the wild type, but none are completely capable of replacing GroEL function. These observations suggest that the phospho-mimetic mutations might have contributed to the gain of chaperonin function, although partially, in the DM variant, resulting in its partial chaperonin activity.

**Fig. 3:**
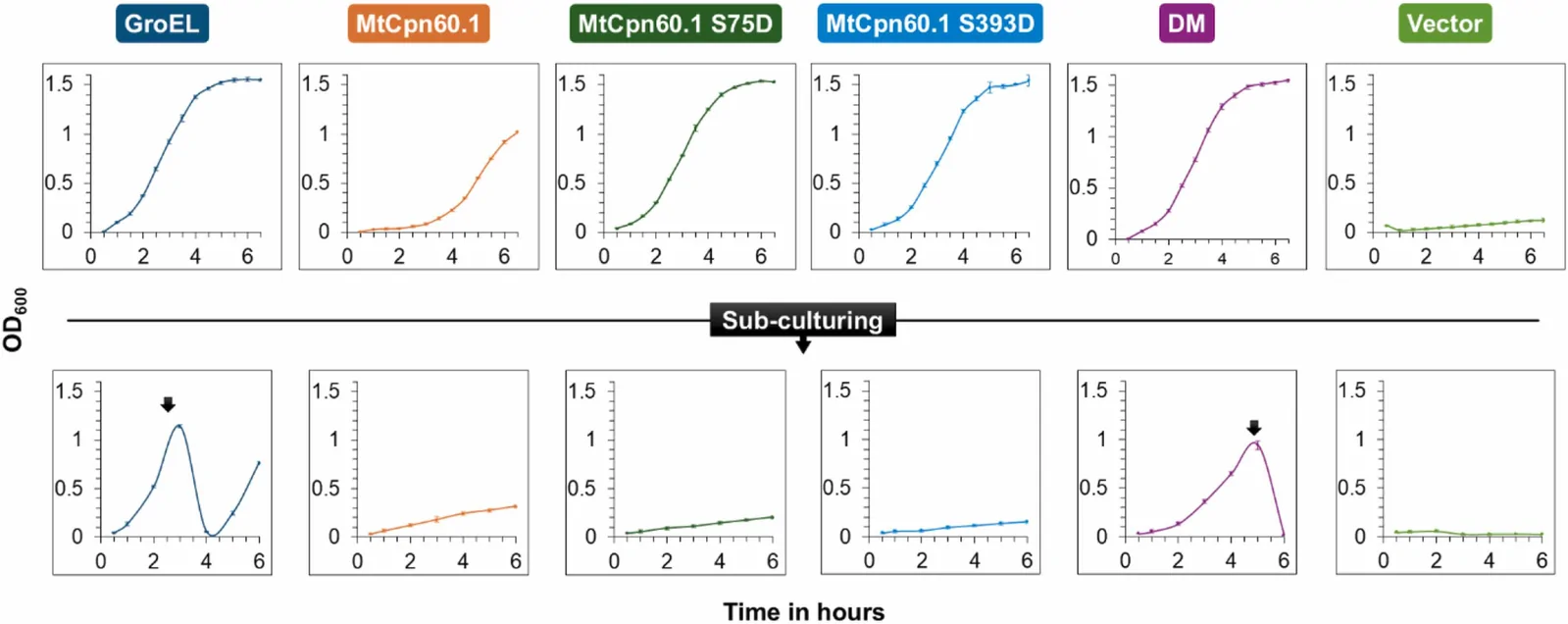
*The DM variant showed partial chaperonin function in restoring growth in liquid cultures. E. coli* MGM100 expressing the indicated chaperonins grown in the presence of 0.2% L-arabinose in a Biomek-i5 workstation were sub-cultured into LB supplemented with 0.2% D-glucose, and their growth was monitored at OD_600_ for 6.5 h. The resultant cultures were further sub-cultured into LB supplemented with 0.2% D-glucose, and their growth was monitored at OD_600_ for a further 6 h. The cultures expressing GroEL and DM variants were further subcultured after crossing the OD of 1.0, as indicated by the filled black arrows. The growth curves are representative of three independent biological repetitions.

### ATPase motif mutations in the double mutant abolish its chaperonin activity

Chaperonin activity requires binding and hydrolysis of ATP, so as a further test of the chaperonin activity of the DM variant, we mutated the conserved residues D51, D86, and D396 (Figure 4a and Supplementary data file 1), corresponding respectively to the ATP-binding residues D52, D87, and D398 of *E. coli* GroEL.^15^, ^16^, ^70^ All the three aspartic acid residues were individually mutated to alanine residues in MtCpn60.1 and the DM variant. The loss of activity following mutation of the ATP-binding residues (Figure 4b) is consistent with ATP-dependent chaperonin activity of the DM variant.

**Fig. 4:**
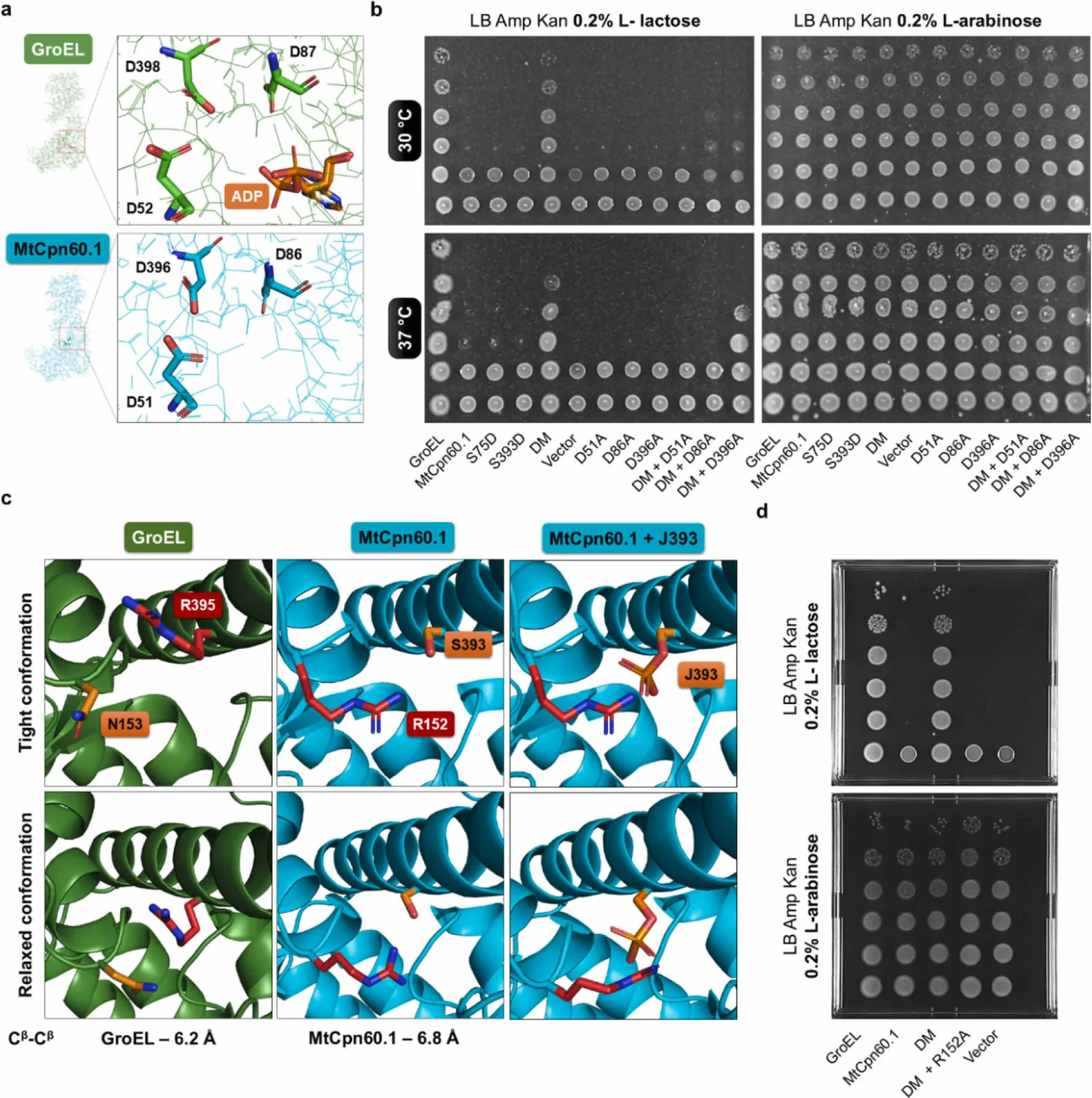
*Mutations affecting ATP-binding and S393 interactions abolish chaperonin activity of the DM variant.***(a)** Alanine-scanning mutagenesis of the ATP binding pocket residues. Molecular diagrams showing the aspartic acid residues in GroEL’s ATP binding pocket (1PF9)^71^ and the corresponding residues in MtCpn60.1. These residues were individually mutated to alanine residues. **(b)** Activity assay of the ATPase mutants. Serially diluted cultures of *E. coli* MGM100 expressing the indicated chaperonin genes were spotted onto the surface of LB agar plates, with supplements as shown. The plates were incubated at the indicated temperatures. S75D and S393D are the respective MtCpn60.1 phospho-mimetic mutants, DM indicates S75D and S393D double mutant. D51A, D86A, and D396A are the respective ATP-binding site mutants. **(c)** Phosphorylation on S393 bridges the gap between the amino acid pair in Cpn60.1. Molecular models of the chaperonin variants in the two conformational states depict the site of phosphorylation (S393). The amino acid residues in the pairs are color-coded, and their sidechains are presented as sticks. Swapping of the indicated residue pair between *E. coli* GroEL (green) and MtCpn60.1 (cyan) can be seen. The molecular distances between the C^β^ carbons in the indicated amino acid pairs are denoted. The letter J indicates a phosphorylated serine. **(d)** R152 – S393 interaction is important in the chaperonin function of MtCpn60.1. Serially diluted cultures of *E. coli* MGM100 expressing the indicated chaperonins were spotted onto the surface of LB agar plates supplemented with L-arabinose (permissive) and L-lactose (restrictive), the plates were incubated at 30 °C. DM indicates the double (S393D/S75D) phospho-mimetic mutant of MtCpn60.1.

### Homology modeling suggests that phosphorylation modulates chaperonin activity via unique positioning of an amino acid pair

To understand the significance of phosphorylation on S393, we modeled MtCpn60.1 using the coordinates of *E. coli* GroEL structure (PDB ID: 1AON).^16^ In MtCpn60.1, the residue S393 corresponds to a conserved arginine, which is found in GroEL (R395) and Cpn60.2 homologues (R393) (Figure S6 and Supplementary data file 1). As phosphorylated S393 would have the opposite charge to the arginine, we scanned the neighboring residues in our models to see whether a charge reversal could lead to a similar salt bridge. We found that a conserved asparagine (N153) in GroEL is substituted by an arginine (R152) in MtCpn60.1 (Figure 4c). Thus, the N153/R395 pair in GroEL could be replaced by an R152/S393 pair in MtCpn60.1, if the serine carried a negatively charged phosphate. S393 and R153 are separate in the primary structure but are quite close in the tertiary structure (Figure S6). Since N153 and R395 have been demonstrated to play important roles in the conformational transitions of GroEL, we examined how the corresponding residues in MtCpn60.1 are positioned in the relaxed and tight conformational states, comparing their positions to the corresponding residues in GroEL. For this, we took the distance between the β-carbon atoms as a measure of the separation in the residue-pair. The β-carbon atom distance between N153 and R395 in *E. coli* GroEL was 6.2 Å and the corresponding distance between in MtCpn60.1 R152 and S393 was 6.8 Å (Figure 4c). The higher separation in MtCpn60.1 does not, however, take account of the size of a phosphate group on the serine residue, and we predict that a phosphate group on S393 would fill this gap. To test this, we added a phosphate group (HPO_3_^−^) onto the side chain oxygen of S393 in MtCpn60.1 structural models and simulated the result using normal mode analysis.^30^, ^35^, ^72^ The resulting models confirmed that the gap could be effectively filled by serine phosphorylation (Figure 4c). To test if the predicted R152/phospho-S393 interaction is important in chaperonin activity, we generated a R152A mutant in the DM variant background. This mutation abolished the DM variant’s chaperonin activity (Figure 4d), consistent with an important role for the residue pair in chaperonin activity.

### The DM variant shows native-PAGE migration consistent with higher-order oligomerization and exhibits partial chaperonin activity

We have previously shown phosphorylation of MtCpn60.1 S393 to be correlated with the formation of a double toroid in *M. tuberculosis*,^43^ but phosphorylation of MtCpn60.1 S75 has not previously been reported. Position S75 corresponds to a conserved glutamic acid (E76) in GroEL that plays a role in oligomerization,^37^, ^61^ consistent with our finding that a phospho-mimetic mutation at this position aids chaperonin activity. Moreover, S75 phosphorylation was predicted by the phosphorylation prediction algorithms NetPhos 3.1^73^ and KinasePhos 2.0^74^ (Figure S3). To confirm whether S75 is indeed phosphorylated in vivo, we cloned *Mtcpn60.1* into a mycobacterial expression vector, pMV261,^75^ and expressed and purified MtCpn60.1 from a *M. smegmatis Δcpn60.1* strain.^45^ Since this strain lacks a resident Cpn60.1, the Cpn60.1 homologue produced was exclusively MtCpn60.1. We digested the purified MtCpn60.1 with the aspartic acid-specific protease AspN,^76^ as tryptic digestion would result in a five-residue peptide H_3_N^+^-^75^SVATK^79^-COO^-^ (Figure S8), which was at the lower detection limit for our mass-spectrometer, while AspN digestion resultx in a 14-residue peptide H_3_N^+^-^68^LGAQLVKSVATKTN^81^-COO^-^. The resulting AspN digest was subjected to tandem mass-spectrometry on the Orbitrap Elite hybrid mass spectrometer and identified four peptides having post translational modifications, three peptides showed seryl phosphorylation, and two peptides showed deamidation (Figure 5a). The phosphopeptides, H_3_N^+^-^67^DLGAQLVKSVATKTN^81^-COO^-^ and H_3_N^+^-^435^ DEVLGVDVFSEALAAPLFWIAANAGL ^460^-COO^-^ contained residues S75 and S444, respectively, that showed strong purifying selection (Figures 1 and S1) and were tested in the complementation assay for their role in oligomerization (Figure 2). As the peptides H_3_N^+^-^67^DLGAQLVKSVATKTN^81^-COO^-^ and H_3_N^+^-^435^ DEVLGVDVFSEALAAPLFW IAANAGL ^460^-COO^-^ contain single serine residues, it is likely that the phosphorylation in these peptides is on S75 and S444, respectively. However, as the phosphopeptide H_3_N^+^-^124^ DAVSEALLASATPVSGKTGIAQVATVSSRDEQIG ^157^-COO^-^ contained to multiple serines, we could not assign the site of phosphorylation.

**Fig. 5:**
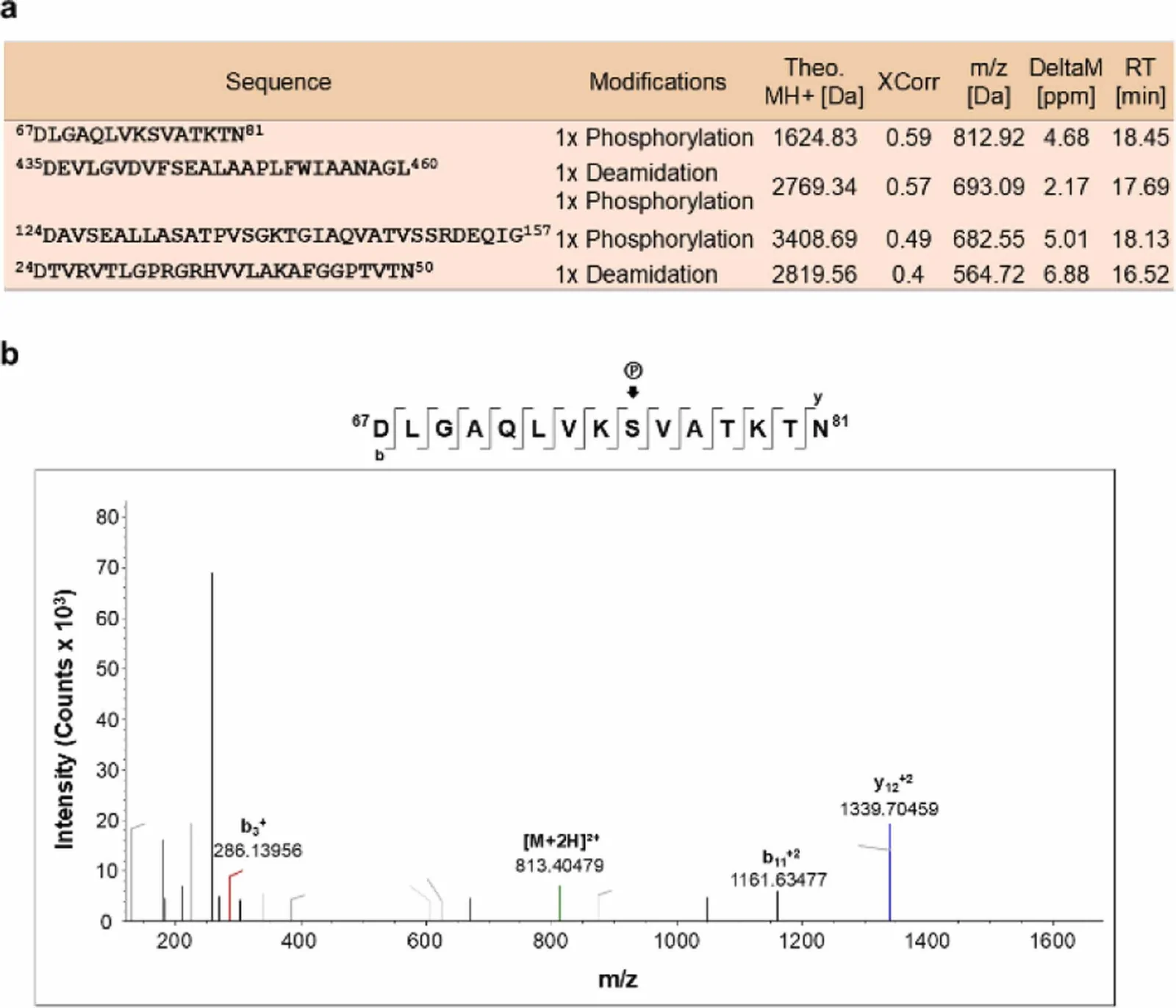
*Phosphorylation of Cpn60.1.***(a)** MtCpn60.1 AspN digest was resolved though LC-MS/MS analysis on the Orbitrap Elite mass spectrometer, and the peptides exhibiting phosphorylation and deamidation with high confidence are listed with their migration parameters. **(b)** Detection of phosphorylation on MtCpn60.1 S75. LC-MS/MS fragmentation analysis of the peptide DLGAQLVKSVATKTN ([MH]+ *m/z* 1624.83058, DeltaM ppm: 4.68), resulting from AspN digest, corresponding to amino acid residues 67–81 of MtCpn60.1. The peaks and potential fragmented ions corresponding to the indicated peptide [y(0) to y(12) and b(0) to b(12)] are labeled.

As the peptide H_3_N^+^-^67^DLG AQLVKSVATKTN^81^-COO^-^ contained S75 residue, we analyzed it further to determine if the phosphorylation is indeed on S75. Analysis of the resulting fragmented ions, y(0) to y(12) and b(0) to b(12), using Proteome Discoverer v22 revealed that the peptide with a theoretical mass (MH+) of 1544.77 Da^77^ exhibited 1624.83 Da MH+, corresponding to the addition of a HPO_3_^−^ ion and a delta M (ppm) of 4.68 (Fig. 5b). This is consistent with the presence of a phosphorylated serine residue in this peptide and additionally with the fact that a threonyl phosphorylation was not observed in MtCpn60.1 isolated from *M. tuberculosis*.^43^ Further, the peak at 1161.63477 *m/z* corresponded to a 10-residue phosphorylated ion, H_3_N^+^-^68^LGAQLVKSVA^77^-COO^-^, that shifted about 80 da from its theoretical molecular mass (1082.620955), consistent with phosphorylation on constituent Serine-75, which is 8th residue in the ion.

We did not detect the phosphorylation on S393, as this position, upon AspN digestion, is in a 35-residue peptide, which is at a significantly higher detection limit for our mass-spectrometer (Figure S8). Phosphorylation on S393 was detected earlier^43^ following tryptic digestion, which resulted in a 11 residue peptide (Figure S8).

Since the chaperonin activity requires the formation of the typical double toroid quaternary structure,^15^, ^28^, ^78^ and the MtCpn60.1 double phospho-mimetic mutant shows a higher level of chaperonin function in vivo, we predicted that it would assemble into oligomers. To test this prediction, as the DM and single mutants were difficult to purify, we tested their migration on native-PAGE. lysates of MGM100 expressing MtCpn60.1 and S393D and DM phospho-mimetic mutants were resolved through 6.5% native-PAGE (Figure S9). *E. coli* lysates expressing GroEL and the functional single-ring variant SR44^79^ were included as markers for double- and single-ring variants. Although neither WT MtCpn60.1 nor MtCpn60.1 S393D showed bands corresponding to either the single or double ring forms of GroEL, the DM variant showed bands in both these positions (Figure S9), suggesting that the migration of DM similar to GroEL on native-PAGE is consistent with phosphorylation-associated higher-order oligomerization. However, definitive evidence of the oligomers’ chaperonin activity would require chaperonin assays using purified oligomeric forms, which is technically challenging as these proteins are highly unstable and difficult to purify.

## Discussion

Although most of our understanding of chaperonin mechanisms is derived from the work on *E. coli* GroEL, not all chaperonins necessarily fit the GroEL paradigm. The fact that many bacteria possess multiple GroEL homologues, in particular the pathogenic bacteria, is consistent with the view that diverse mechanisms might have arisen that determine chaperonin structure and function, which could even coexist in the same cell.^3^, ^8^ Mycobacteria were the first bacterial genus to be identified with multiple chaperonin homologues,^31^ and this, together with reports of the moonlighting properties of their chaperonins and the fact that evolution rates of the Mycobacterial chaperonins differ, are of particular interest in this regard.^10^, ^62^ Notably, only one of the two chaperonin genes, *cpn60.2*, is essential,^6^, ^40^, ^41^ but loss of the non-essential mycobacterial chaperonin, *cpn60.1*, leads to several changes in phenotype, including loss of the ability of *M. tuberculosis* to form granulomas following infection of mice and guinea pigs,^6^ alteration of the cell envelope and biofilm formation in both *M. bovis*^46^ and *M. smegmatis*,^45^ and metabolic changes in *M. bovis*.^6^, ^46^ These observations have led to the proposal that Cpn60.2 functions as a general housekeeping chaperonin, but Cpn60.1 has a more specialized function with a role in pathogenesis.^8^, ^80^ Mycobacterial Cpn60.1 proteins generally fail to complement the loss of *groEL* in *E. coli*, and do not have the oligomeric structure typical of GroEL and other chaperonins, which is needed for the formation of the protected areas (Anfinsen cages) wherein the protein clients are thought to fold.

Following our earlier work showing that phosphorylation of MtCpn60.1 at S393 correlates with its oligomerization into a tetradecamer and limited chaperonin activity,^42^, ^43^ we here show that S75 phosphorylation can be detected in vivo and that the combined S75D/S393D phospho-mimetic substitutions confer partial complementation of GroEL function in *E. coli* (Figures 2 and 3). Phosphorylation of both residues appears to be necessary for maximal activity (Figure 2). Similarly, mutations in the putative ATP-binding residues of MtCpn60.1 resulted in the loss of chaperonin activity (Figure 4). As ATPase activity is essential for GroEL chaperonin activity,^15^, ^16^, ^70^ the mutations in the ATP-binding motif would have led to the loss of function (Figure 4). Since the ATP-binding site mutations do not interfere with chaperonin assembly into a tetradecamer,^15^, ^16^, ^70^ this observation is consistent with the hypothesis that the loss of activity in these mutants is likely to be due to failure of the oligomeric Cpn60.1 to complete the multiple rounds of ATP hydrolysis needed for chaperonin activity. These findings, together with the sequence (Figure 1 and Supplementary data file 1) and structural^16^, ^50^, ^52^, ^81^ comparisons of the Mycobacterial chaperonin paralogues, are consistent with the hypothesis that specific phosphorylation events are likely to restore inter-subunit salt bridges in MtCpn60.1, which are likely to stabilize the oligomeric forms.

Even the DM variant was not fully functional in the complementation studies, as it failed to replace *groEL* in the deletion strain AI90 and to complement at elevated temperatures in the *groESL* conditional mutant strain, MGM100 (Figure 2). Residual GroEL activity under the repressing conditions (Table S3) was higher in LG6^82^ than in MGM100^67^ and may thus be required for complementation under more stringent conditions. Further, the DM variant could not rescue the culture in the absence of the inducer (Figure 3). These observations suggest that the DM variant requires some GroEL to be present for either for its own folding or assembly, a role that could be replaced by MtCpn60.2 in the native host, *M. tuberculosis*. As some bacteria expressing multiple functional chaperonins form mixed oligomers under certain conditions,^83^ the presence of DM may enable the formation of mixed oligomers with resident GroEL. These DM-GroEL mixed oligomers may likely support the cellular protein folding. In the absence of the DM, the resident GroEL (expressed in restricted conditions) would not make enough oligomers to support cellular protein folding. Alternatively, incomplete oligomerization of the DM leading to a relatively smaller amount of active chaperonin, sub-optimal activity of the assembled oligomer, and/or sub-optimal chaperonin-client protein interactions or expression in the strain AI90 might have contributed to the variable activity of DM. The observation that the DM mutant complemented LG6, while wild type MtCpn60.1 could not, suggests that the phosphorylation on two residues might have triggered phosphorylation-associated higher-order oligomerization and contributed to the partial chaperonin activity of the DM variant (Figures 2 and 3).

To understand the molecular basis for the observed effect of the phospho-serines and the phospho-mimetic mutants, in lieu of crystal structures for this region of MtCpn60.1, we generated homology models of MtCpn60.1, in relaxed and tight conformations, to provide clues to answering this question. The consequence of phosphorylation on S75 resulting in MtCpn60.1 chaperonin activity is comprehendible as S75 corresponds to a conserved Glutamic acid in GroEL and several double toroid-forming essential chaperonin homologues (Supplementary data file 1), which was demonstrated to be involved in oligomerization by forming salt bridges with the neighboring subunits.^16^, ^61^ Therefore, serine at this position in MtCpn60.1 abolished the salt bridges and oligomeric assembly.^37^, ^42^, ^43^, ^52^ These observations are consistent with the possibility that the S75D substitution promotes oligomerization (Figure 2c and d); however, S75D alone is inactive, and the S75D/S393D double mutant remains only partially functional.

Further, a critical residue pair switching was observed between R395/N153 in GroEL and S393/R152 in MtCpn60.1 homology models (Figure 4). The residue S393 was proximally placed with R152 in Cpn60.1, similar to residues R395/N153 of GroEL, in the tight conformation. However, the residues move apart in Cpn60.1 in the relaxed conformation (Figure 4c), and we propose that phosphorylation (or the presence of a phospho-mimetic residue S393 can bridge the larger gap and restore the inter-subunit contacts. R395 in *E. coli* GroEL also forms an essential intra-subunit salt bridge with D155.^14^, ^84^ Breaking this R395-D155 salt bridge with a D155A mutation causes a range of adverse effects on GroEL, including loss of positive cooperativity within the ring by altering the concerted conformational transitions to sequential transitions,^85^ weakening a proximal inter-subunit salt bridge R197-E386 resulting in stabilization of an intermediate with 3–4 subunits in tight conformation and the others in the relaxed conformation,^85^ converting the mono-sigmoidal ATP-dependency of chaperonin activity a to bi-sigmoidal dependency^86^ and affecting the 3D position of D398, which is responsible for providing water for γ-phosphate of ATP.^16^, ^71^, ^87^ These observations indicate the importance of this salt bridge on the allostery and activity of GroEL. This arginine residue exhibits a distinct pattern of conservation in mycobacterial chaperonins (Table 1 and Supplementary data file 1); it is conserved among the Cpn60.2 paralogues but is substituted by serine in the Cpn60.1 paralogues. This could provide a mechanism whereby a Mycobacterial cell could control the activity of Cpn60.1 by differentially phosphorylating this residue. Interestingly, Cpn60.1 paralogues from rapidly growing mycobacteria such as *M. smegmatis*, *M. goodie*, and *M. chubuense*^88^ that are phylogenetically distinct (Figure 1 and Table 1) have alanine instead of serine at this position. This rules out any phosphorylation-mediated regulation via the mechanism discussed above, so Cpn60.1 in these mycobacteria might be inactive as a chaperonin and have a different role.

In this context, it is also intriguing to note that the Cpn60.1 paralogues from rapid- and slow-growing mycobacteria differ at residue 28 (Supplementary data file 1), which is located adjacent to the ATP binding pocket and is involved in ATP-dependent interaction with V94.^89^ Similar to *E. coli* GroEL and mycobacterial Cpn60.2 paralogues, the Cpn60.1 paralogues from rapidly growing mycobacteria have arginine at this position, while the Cpn60.1 paralogues from slow-growing mycobacteria have lysine (Supplementary data file 1), suggesting that the interaction with the nucleotide might be different between these two types of Cpn60.1 paralogues. Taken together, these observations are consistent with the hypothesis that the non-essential Cpn60.1 paralogues of the rapidly growing and slow-growing mycobacteria might have diverged in their mechanisms and cellular functions: further work will be required to test this idea.

We detected a phosphorylated peptide containing S444 (Figure 5a). However, our MS analysis did not definitively localize the modification to S444. The S444D phospho-mimetic variant was non-functional in the complementation assay (Figure 2b), and therefore these data do not support a role for S444 phosphorylation in the oligomerization phenotype examined here. Therefore, we did not investigate this mutation here; however, more investigation is needed to determine its function if it is linked to MtCpn60.1’s in vivo or ex vivo (pathogenic) functions.

Purified MtCpn60.1 from *M. tuberculosis* has previously been demonstrated to be phosphorylated in vitro by the ser/thr protein kinase from *M. tuberculosis*, PknF. In this study, phosphorylation was shown to occur at two threonine positions, T25 and T54.^60^ It was also shown that Cpn60.1 from *M. smegmatis* was not a substrate for this kinase, another demonstration that Cpn60.1 paralogues from different Mycobacteria do not necessarily behave in the same way or have similar cellular roles. The discrepancy between the phosphorylated residues detected in the current study and previous in vitro work^60^ could arise from the different experimental conditions (*in vivo* vs *in vitro*), and we cannot exclude the possibility that the phosphorylation pattern of *M. tuberculosis* Cpn60.1 is different depending on whether it is expressed in its native strain or in *M. smegmatis*, though the consistent results of the MS and genetic experiments means this seems unlikely.

In summary, utilizing genetic, biochemical, structural, and bioinformatics approaches, we show that the phosphorylation of MtCpn60.1 is likely to associated with its higher-order oligomerization and acquisition of partial chaperonin activity. Mycobacterial Cpn60.1 homologues have been demonstrated to be involved in different stages of pathogenesis, such as granuloma formation and several aspects of immune cell signaling.^6^, ^47^, ^48^, ^90^ Therefore, understanding the role of phosphorylation in these functions and identifying the kinases (or kinase cascade) involved would unravel the potential temporal regulation of the two distinct functions of Cpn60.1’s, the chaperonin activity (protein folding) and pathogenic roles, during the active- and latent-TB disease stages. This further can enhance our understanding of the disease and treatment options and on the client-protein preferences between the chaperonin paralogues Cpn60.1 ad Cpn60.2. We propose that phosphorylation-mediated oligomerization may be a way in which mycobacteria can control the activity of Cpn60.1 homologues, and this may be an important aspect of its role in pathogenesis.

## Materials and methods

### Complementation assays

Complementation assays were done as reported earlier.^30^, ^35^, ^43^, ^91^ Briefly, strains LG6 and SV2 were transformed with pBAD24-derived expression plasmids, while strains MGM100 and AI90 were transformed with pTrc99A-derived expression plasmids (Table S2). Stationary phase cultures of *E. coli* expressing phospho-mimetic *cpn60.1* variants were serially diluted and spotted onto LB agar plates for permissive and restrictive conditions (Table S3). One set of the plates was incubated at 30 °C and one at 42 °C. The complementation ability of the ATP-binding site variants was assessed in MGM100 at 30 °C and 37 °C.

### In vivo assay for bacteriophage morphogenesis

Bacteriophage morphogenesis was assessed as reported earlier.^43^ Briefly, LG6 expressing different phospho-mimetic Cpn60.1 variants was cultured in LB supplemented with 0.4% D-maltose, 5 mM MgCl_2_, and 5 mM CaCl_2_, 1 mM IPTG, and the appropriate antibiotic. Stationary phase cultures were overlaid in soft agar onto LB plates containing either 0.2% D-glucose, 0.2% L-arabinose, or 1 mM IPTG. The plaque-forming ability of bacteriophages P1, λ_*c1B2*_, and T4GT7 was assessed by spotting 5 μl each of hundred-fold diluted suspension of bacteriophage stocks onto these plates. The plates were incubated at 30 °C and assessed visually after overnight incubation.

### Analysis of oligomeric status of MtCpn60.1 using native page

MGM100 cells expressing Cpn60.1 phospho-mimetic variants were suspended in lysis buffer I (50 mM HEPES pH: 7.5, 150 mM NaCl, 1 mM EDTA, 1 mM PMSF, and 10 mM Sodium lauryl sarcosine), transferred to Lysing Matrix B (MP Biomedicals, Inc.), and lysed in FastPrep-24™ 5G Instrument (MP Biomedicals Inc.), using the built-in *E. coli* lysing protocol. The soluble proteins were recovered by centrifugation, resolved through 6.5% Native PAGE, and stained with Coomassie brilliant blue (CBB). *E. coli* MGM100 expressing WT GroEL^43^ and a single ring variant^79^ were included as markers.

### Detection of phosphorylation on S75D

*Mtcpn60.1* was cloned into *Mycobacteria*-*E. coli* shuttle vector, pMV261^75^ to generate pMV60.1 and the resulting plasmid was used to transform an *M. smegmatis cpn60.1* deletion strain,^45^ so that the Cpn60.1 homologue produced will be exclusively the plasmid-borne copy. The resulting strain was cultured in Middlebrook 7H9 supplemented with 10% OADC (Difco Laboratories, Fisher Scientific, UK) at 37 °C. Since phosphorylation on Cpn60.1 was enhanced at 42 °C,^43^ the cultures were shifted to 42 °C at the late-log phase (OD_600_ = 1.0) and incubated for 4 h. Cells were recovered by centrifugation and resuspended in lysis buffer II (50 mM Tris.HCl pH: 7.5, 150 mM NaCl and protease inhibitor cocktail (Roche Holding AG, Basel, Switzerland)), transferred to Lysing Matrix E (MP Biomedicals, Inc.), and lysed in FastPrep-24™ 5G Instrument (MP Biomedicals Inc.), using the built-in Mycobacterial lysing protocol. The lysates were loaded onto Ni-NTA Spin Columns (Qiagen Inc., Hilden, Germany) and eluted with the elution buffer (50 mM Tris.HCl pH 7.5, 150 mM NaCl, and 40 mM Imidazole). The purified protein was resolved on 12.5% SDS-PAGE^91^ and stained in CBB. The bands corresponding to Cpn60.1 were excised and analyzed for evidence of phosphorylation by the University of Birmingham Advanced Mass Spectrometry Facility, Birmingham, UK. Briefly, the protein was digested using AspN protease (Promega Corporation, Madison, WI, USA), following the manufacturer’s instructions. Phosphorylated peptides were enriched using FeIMAC and were subjected to LC-MS/MS analysis on Orbitrap Elite hybrid mass spectrometer (Thermo Fisher Scientific, UK). The raw data were analyzed using the Proteome Discoverer application 2.2 (Thermo Fisher Scientific, UK).

## Funding and support

This work was supported by grants from the Biotechnology and Biological Sciences Research Council (BBSRC), UK (BB/S017526/1), the Royal Society, UK (Newton International Fellowship - NF161469), and the Department of Biotechnology, India (Sys-TB project - BT/PR3260/BRB/10/967/2011).

## CRediT authorship contribution statement

**C. M. Santosh Kumar:** Writing – original draft, Methodology, Investigation, Conceptualization. **Monika Singh:** Data curation. **Shekhar C. Mande:** Writing – review & editing, Supervision. **Peter A. Lund:** Writing – review & editing, Supervision.

## Declarations of interest

The authors declare that they have no known competing financial interests or personal relationships that could have appeared to influence the work reported in this paper.

## Data Availability

All data used in this article are presented in the supplementary information.

## Footnotes

Appendix A Supplementary data associated with this article can be found in the online version at doi:10.1016/j.cstres.2026.100220.
