# Supplementary material for: Seryl phosphorylation modulates chaperonin function of Mycobacterial chaperonin, Cpn60.1
Source: Cell Stress Chaperones. 2026 Sep 25;31(6):100220. doi: 10.1016/j.cstres.2026.100220 (PMC13673040; doi:10.1016/j.cstres.2026.100220)
Supplement: Supplementary file 1 [file mmc1.docx]

**Supplementary Information**

**for**

**Seryl phosphorylation modulates chaperonin function of Mycobacterial Chaperonin, Cpn60.1**

**C. M. Santosh Kumar^1^*, Monika Singh^2^, Shekhar C. Mande^3,4^, Peter A. Lund^1^.**

^1^ School of Biosciences & Institute of Microbiology and Infection, University of Birmingham, Birmingham, UK B15 2TT.

^2^ Laboratory of Structural Biology, Centre for DNA Fingerprinting and Diagnostics, Hyderabad, India. 500001.

^3^ Laboratory of Structural Biology, National Centre for Cell Science, Pune, India. 411007.

^4^Current Affiliation: Bioinformatics Centre, Savitribai Phule Pune University, Pune, India 411007

***Corresponding author:** **C M Santosh Kumar** s.k.cm@bham.ac.uk

**Running title:** PTMs Drive Activity of Cpn60.1.

**Supplementary Methods**

**Strains, plasmids and growth conditions.** Complementation studies of the chaperonin variants were done in four *groESL* mutant *E. coli* strains. The first two strains were (1) LG6, in which the chromosomal copy of the *groESL* operon is expressed from the lactose or IPTG inducible *P_lac_* promoter and thus the strain cannot grow in the absence of lactose or IPTG [1], and (2) MGM100, in which the chromosomal copy of the *groESL* operon is expressed from the L-arabinose inducible *P_BAD_* promoter and thus the strain cannot grow in the absence of L-arabinose [2]. In both these strains, growth on non-permissive media requires expression of a complementing chaperonin. In the third strain, AI90, the *groEL* gene is replaced with a Kan^R^ cassette. Growth of this strain requires the presence of a complementing chaperonin gene under all conditions, and in the experimental strain this provided on a Cam^R^ shelter plasmid [3]. The fourth strain SV2 harbours a temperature sensitive *groEL44* allele and thus is unable to grow at high temperature unless complemented by a functional GroEL [4]. The plasmid vectors, oligonucleotide primers and strain-specific growth conditions employed in this study are listed in tables S1, S2 and S3, respectively. All the chemicals, media supplements, molecular biology reagents and kits were purchased from Sigma Inc., HiMedia Laboratories Pvt. Ltd., New England BioLabs and Qiagen Inc., respectively.

**Sire directed mutagenesis**. All mutations were constructed using QuikChange Lightning Site-Directed Mutagenesis Kit (Agilent Technologies, Santa Clara, CA, USA) using the plasmids and primers listed in tables S1 and S2, respectively.

**Homology modelling of *M. tuberculosis* Cpn60.1.** *M. tuberculosis* Cpn60.1 sequence was modelled using the co-ordinates from the *E. coli* GroEL structure (PDB ID: 1AON) [5] for the chains A and H that represent the **R** and **T** conformational states, respectively. Five models were generated for each conformation using Modeller 9.13 [6] and the model with the lowest energy was selected. C^β^ distances between the residues were measured using PyMol molecular visualization application 2.0.A phosphate group (HPO_3_^−^) was built on to the side-chain oxygen of S393 in the Cpn60.1 models and the models were simulated using normal mode analysis [7; 8].

**Phylogenetic analysis of mycobacterial chaperonin proteins.** Phylogenetic analysis of the mycobacterial chaperonins was performed as reported earlier [9; 10]. Briefly, 58 polypeptide sequences of mycobacterial Cpn60.1 and Cpn60.2 homologues were obtained from UniProt and the sequences were aligned to *E. coli* GroEL using MUSCLE [11], a phylogenetic analysis using the Neighbour-Joining method was inferred with five hundred rounds of boot strapping in MEGA 6.0 [12] and the phylogenetic tree was constructed using FigTree v1.4.4 (http://tree.bio.ed.ac.uk/ software/figtree/). Since the Cpn60.1 paralogues from *M. gilvum* and *M. vanbaalenii* bear a Gly-Met rich carboxy terminus and consequently have been branching with the Cpn60.2 paralogues from other mycobacteria and likewise, Cpn60.2 paralogues of these organisms bear a His-rich carboxy terminus and have been branching with Cpn60.1 from other mycobacteria. Therefore, we have swapped the annotations for these chaperonins for clarity (Fig. 1A and Supplementary data file 1). The polypeptide and corresponding nucleotide sequence alignments were used in estimating the conservation (using ConSurf Rate4Site [13]) and rate of evolution (using SelectOn [14] and MEGA11 [12]).

**Growth curves.** The growth of *E. coli* MGM100 expressing different chaperonin variants was monitored in a Biomek-i5 workstation. The initial cultures were grown in the presence of 0.2% L-arabinose and sub-cultured into LB supplemented with 0.2% D-glucose and their growth was monitored at OD_600_ for 6.5 hours. The resultant cultures were further sub-cultured into LB supplemented with 0.2% D-glucose and their growth was monitored at OD_600_ for a further 6 hours. Cultures at this stage were programmed to be sub-cultured after reaching an OD_600_ of 1.0. Three cultures for each strain were monitored.

**Supplementary Tables**

**Table S1.** Plasmid vectors used in this study.

| Plasmid | Description | Source |
| --- | --- | --- |
| pBAD24 | Arabinose inducible expression vector | [15] |
| pTrc99A | Lactose or IPTG inducible expression vector | [16] |
| pMV261 | Mycobacterial expression vector | [17] |
| pBADGSL | *Ec groESL* operon cloned in pBAD24 | Lab stock |
| pTrcGSL | *Ec groESL* operon cloned in pTrc99A | Lab stock |
| pBAD60.1 | *Mtcpn10* + *Mtcpn60.1* cloned in pBAD24 as operon | Lab Stock |
| pTrc60.1 | *Mt cpn10* + *Mtcpn60.1* cloned in pTrc99a as operon | Lab Stock |
| pBAD60.1 S75D | S75D mutation in *Mtcpn60.1* in pBAD60.1 |  |
| pBAD60.1 S393D | S393D mutation in *Mtcpn60.1* in pBAD60.1 |  |
| pBAD60.1 S416D | S416D mutation in *Mtcpn60.1* in pBAD60.1 |  |
| pBAD60.1 S444D | S444D mutation in *Mtcpn60.1* in pBAD60.1 |  |
| pBAD60.1 S484D | S484D mutation in *Mtcpn60.1* in pBAD60.1 |  |
| pBAD60.1 S75D S393D (DM) | S75D mutation in *Mtcpn60.1* in pBAD60.1 S393D |  |
| pBAD60.1 S393D S416D | S416D mutation in *Mtcpn60.1* in pBAD60.1 S393D |  |
| pBAD60.1 S393D S444D | S444D mutation in *Mtcpn60.1* in pBAD60.1 S393D |  |
| pBAD60.1 S393D S484D | S484D mutation in *Mtcpn60.1* in pBAD60.1 S393D |  |
| pTrc60.1 S75D | S75D mutation in *Mtcpn60.1* in pTrc60.1 |  |
| pTrc60.1 S393D | S393D mutation in *Mtcpn60.1* in pTrc60.1 |  |
| pTrc60.1 S75D S393D (DM) | S75D mutation in *Mtcpn60.1* in pTrc60.1 S393D |  |
| pMV60.1 | *Mtcpn60.1* cloned into pMV261 |  |
| pTrc60.1 D51A | D51A mutation in *Mtcpn60.1* in pTrc60.1 |  |
| pTrc60.1 D86A | D86A mutation in *Mtcpn60.1* in pTrc60.1 |  |
| pTrc60.1 D396A | D398A mutation in *Mtcpn60.1* in pTrc60.1 |  |
| pTrc60.1 DM D51A | D51A mutation in *Mtcpn60.1* in pTrc60.1 DM |  |
| pTrc60.1 DM D86A | D86A mutation in *Mtcpn60.1* in pTrc60.1 DM |  |
| pTrc60.1 DM D396A | D398A mutation in *Mtcpn60.1* in pTrc60.1 DM |  |

* Unless mentioned, the source of the plasmid vectors is this study.

**Table S2.** Oligonucleotide primers used in this study.

| **Name** | **Sequence** | **Source*** |
| --- | --- | --- |
| **pBAD For** | 5’ CTGTTTCTCCATACCCGTT 3’ | [15] |
| **pBAD Rev** | 5’ CTCATCCGCCAAAACAG 3’ | [15] |
| **S75DF** | 5’ CAGCTTGTGAAGGATGTGGCCACCAAGA 3’ |  |
| **S75DR** | 5’ TCTTGGTGGCCACATCCTTCACAAGCTG 3’ |  |
| **S393DF** | 5’ GCAAGGAAGATGTCGAGGATGCGGTC 3’ |  |
| **S393DR** | 5’ GACCGCATCCTCGACATCTTCCTTGC 3’ |  |
| **S416DF** | 5’ GGGGGAGCCGATCTCATCCACCAG 3’ |  |
| **S416DR** | 5’ CTGGTGGATGAGATCGGCTCCCCC 3’ |  |
| **S444DF** | 5’ GACGTGTTCGATGAAGCCCTTGCCG 3’ |  |
| **S444DR** | 5’ CGGCAAGGGCTTCATCGAACACGTC 3’ |  |
| **S484DF** | 5’ ACGTGAATACCCTGGATTATGGTGACTTGGC 3’ |  |
| **S484DR** | 5’ GCCAAGTCACCATAATCCAGGGTATTCACGT 3’ |  |
| **D51AF** | 5’ GGACCCACGGTTACCAACGCGGGCGTCACGGTGGCA 3’ |  |
| **D51AR** | 5’ TGCCACCGTGACGCCCGCGTTGGTAACCGTGGGTCC 3’ |  |
| **D86AF** | 5’ AACGATGTGGCCGGTGCAGGCACCACCACC 3’ |  |
| **D86AR** | 5’ GGTGGTGGTGCCTGCACCGGCCACATCGTT 3’ |  |
| **D396AF** | 5’ AAGGAAGATGTCGAGGCAGCGGTCGCGGCC 3’ |  |
| **D396AR** | 5’ GGCCGCGACCGCTGCCTCGACATCTTCCTT 3’ |  |

* Unless mentioned, the source of the oligonucleotide primers is this study.

**Table S3. Growth conditions employed for *E. coli* *groEL* mutant strains.**

| **Strain** | **Permissive condition** | **Restrictive condition** |
| --- | --- | --- |
| *E. coli* LG6 | LB + 1 mM IPTG | LB + 0.2% L-arabinose |
| *E. coli* MGM100 | LB + 0.2% L-arabinose | LB + 0.2% D-lactose |
| *E. coli* AI90 | LB + 0.2% D-lactose | LB + 0.2% D-lactose + 25 µg/mL Chloramphenicol |
| *E. coli* SV2 | Incubation at 30 °C | Incubation at 42 °C |

**Supplementary figures and captions**

**Figure S1. Conservation profiles of Cpn60.1 and Cpn60.2 homologues**. **a)** Site-specific divergence of Cpn60.1 and Cpn60.2 homologues was mapped onto GroEL sequence. Cpn60.1 homologues exhibit greater divergence than the Cpn60.2 homologues. **b)** Scatter plot comparing the conservation scores of the indicated chaperonin homologues. **c)** The ConSurf scores of Cpn60.1 and Cpn60.2 homologues were mapped onto GroEL sequence. The letters b, e, f and s denote the predicted buried or exposed, functional and structural residues, respectively.

**Figure S2. Conserved sequence from the Multiple sequence alignment of Mycobacterial chaperonins.** The multiple sequence alignment of Mycobacterial chaperonins was input into the EMBOSS Cons and the conserved sequence was obtained using default parameters. The residue positions investigated in this study are highlighted in cyan.

**Figure S3. Prediction of Phosphorylation at S75 in MtCpn60.1.** Polypeptide sequence of MtCpn60.1 was input into the NetPhos 3.1 **(a)** and KinasePhos 2.0 **(b)** phosphorylation prediction servers. **a)** The plot depicts the phosphorylation potential as a function of the amino acid positions and the table lists the predicted positions with the phosphorylation potential score. Position S75, S393, S444 and S484 are highlighted in blue. **b)** Typical output from the KinasePhos 2.0 listing the predicted phosphorylation positions with Hidden Markov model bit scores and sequence logos, which depict the potential phosphorylation. The predicted sites are mapped onto the protein model. Arrows indicate the sites explored in this study. Phosphorylation on S393 was predicted by multiple models, some examples are listed here.

**Figure S4. Locations of the phosphorylation sites on MtCpn60.1 cis and trans ring-forming oligomers.** The S75 and S393 phosphorylation sites were mapped onto MtCpn60.1 *cis* and *trans* ring models.

**Figure S5. The DM variant could Rescue *T_S_* Phenotype of *E. coli* SV2.** Serially diluted cultures of *E. coli* SV2 expressing the indicated chaperonins were spotted onto the surface of LB agar plates and the plates were incubated at the indicated permissive (30 °C) and restrictive (42 °C) temperatures. S393D and DM indicate the respective MtCpn60.1 phospho-mimetic mutants.

**Figure S6. Residue pair swapping in chaperonin sequences.** Multiple sequence alignment of *M. tuberculosis* Cpn60.1 (MtCpn60.1), Cpn60.1 (MtCpn60.2) and *E. coli* GroEL (GroEL) polypeptide sequences. Arrows indicate the positions of MtCpn60.1 N152 and S393 amino acids.

**Figure S7. Potential residue-swapping at MtCpn60.1 S393 site.** Wireframe models showing the relaxed and tight conformations of GroEL and MtCpn60.1. The amino acid pairs N153-R395 (in GroEL) and R152-S393 (in MtCpn60.1) are shown as color-coded sticks.

**Figure S8. Protease digestion patterns of MtCpn60.1.** Potential cleavage sites of Trypsin (blue broken lines) and aspartic acid-specific AspN (red filled lines) and resultant peptides (PeptideCutter program, ExPASy) are mapped onto the MtCpn60.1 sequence. Potential sites of phosphorylation are highlighted in cyan.

**Figure S9. Native PAGE suggests the higher order oligomerization of MtCpn60.1 DM variant.** *E. coli* MGM100 lysates expressing the indicated chaperonin homologues were resolved through 6.5% native PAGE. The filled and hollow arrows in the GroEL and SR44 lanes show the positions of the potential tetradecameric (double ring) and heptameric (single ring) chaperonin forms, respectively. SR44 is an active single ring-forming variant of GroEL, while S393D and DM indicate the respective MtCpn60.1 phospho-mimetic mutants. **References**

[1] A.L. Horwich, K.B. Low, W.A. Fenton, I.N. Hirshfield, and K. Furtak, Folding in vivo of bacterial cytoplasmic proteins: role of GroEL. Cell 74 (1993) 909-17.

[2] N. McLennan, and M. Masters, GroE is vital for cell-wall synthesis. Nature 392 (1998) 139.

[3] A. Ivic, D. Olden, E.J. Wallington, and P.A. Lund, Deletion of Escherichia coli groEL is complemented by a Rhizobium leguminosarum groEL homologue at 37 degrees C but not at 43 degrees C. Gene 194 (1997) 1-8.

[4] K. Tilly, and C. Georgopoulos, Evidence that the two Escherichia coli groE morphogenetic gene products interact in vivo. J Bacteriol 149 (1982) 1082-8.

[5] Z. Xu, A.L. Horwich, and P.B. Sigler, The crystal structure of the asymmetric GroEL-GroES-(ADP)7 chaperonin complex. Nature 388 (1997) 741-50.

[6] N. Eswar, B. Webb, M.A. Marti-Renom, M.S. Madhusudhan, D. Eramian, M.Y. Shen, U. Pieper, and A. Sali, Comparative protein structure modeling using Modeller. Curr Protoc Bioinformatics Chapter 5 (2006) Unit 5 6.

[7] K. Suhre, and Y.H. Sanejouand, ElNemo: a normal mode web server for protein movement analysis and the generation of templates for molecular replacement. Nucleic Acids Res 32 (2004) W610-4.

[8] N. Chilukoti, C.M. Kumar, and S.C. Mande, GroEL2 of Mycobacterium tuberculosis Reveals the Importance of Structural Pliability in Chaperonin Function. J Bacteriol 198 (2016) 486-97.

[9] S.C. Mande, and S.C.M. Kumar, Functional Diversity in Mycobacterial Chaperonins: The Generalists and the Specialists. in: C.M.S. Kumar, and S.C. Mande, (Eds.), Prokaryotic Chaperonins: Multiple Copies and Multitude Functions, Springer Singapore, Singapore, 2017, pp. 67-80.

[10] C.M. Kumar, S.C. Mande, and G. Mahajan, Multiple chaperonins in bacteria--novel functions and non-canonical behaviors. Cell Stress Chaperones 20 (2015) 555-74.

[11] R.C. Edgar, MUSCLE: multiple sequence alignment with high accuracy and high throughput. Nucleic Acids Res 32 (2004) 1792-7.

[12] K. Tamura, G. Stecher, D. Peterson, A. Filipski, and S. Kumar, MEGA6: Molecular Evolutionary Genetics Analysis version 6.0. Mol Biol Evol 30 (2013) 2725-9.

[13] I. Mayrose, D. Graur, N. Ben-Tal, and T. Pupko, Comparison of site-specific rate-inference methods for protein sequences: empirical Bayesian methods are superior. Mol Biol Evol 21 (2004) 1781-91.

[14] A. Doron-Faigenboim, A. Stern, I. Mayrose, E. Bacharach, and T. Pupko, Selecton: a server for detecting evolutionary forces at a single amino-acid site. Bioinformatics 21 (2005) 2101-3.

[15] L.M. Guzman, D. Belin, M.J. Carson, and J. Beckwith, Tight regulation, modulation, and high-level expression by vectors containing the arabinose PBAD promoter. J Bacteriol 177 (1995) 4121-30.

[16] E. Amann, B. Ochs, and K.J. Abel, Tightly regulated tac promoter vectors useful for the expression of unfused and fused proteins in Escherichia coli. Gene 69 (1988) 301-15.

[17] C.K. Stover, V.F. de la Cruz, T.R. Fuerst, J.E. Burlein, L.A. Benson, L.T. Bennett, G.P. Bansal, J.F. Young, M.H. Lee, G.F. Hatfull, and et al., New use of BCG for recombinant vaccines. Nature 351 (1991) 456-60.
